# Efficient Mining of Variants From Trios for Ventricular Septal Defect Association Study

**DOI:** 10.3389/fgene.2019.00670

**Published:** 2019-08-08

**Authors:** Peng Jiang, Yaofei Hu, Yiqi Wang, Jin Zhang, Qinghong Zhu, Lin Bai, Qiang Tong, Tao Li, Liang Zhao

**Affiliations:** ^1^Precision Medicine Research Center, Taihe Hospital, Hubei University of Medicine, Shiyan, China; ^2^School of Computing and Electronic Information, Guangxi University, Nanning, China

**Keywords:** trio-sequencing, k-mer filtering, variant calling, ventricular septal defect, association study, long non-coding RNA

## Abstract

Ventricular septal defect (VSD) is a fatal congenital heart disease showing severe consequence in affected infants. Early diagnosis plays an important role, particularly through genetic variants. Existing panel-based approaches of variants mining suffer from shortage of large panels, costly sequencing, and missing rare variants. Although a trio-based method alleviates these limitations to some extent, it is agnostic to novel mutations and computational intensive. Considering these limitations, we are studying a novel variants mining algorithm from trio-based sequencing data and apply it on a VSD trio to identify associated mutations. Our approach starts with irrelevant *k*-mer filtering from sequences of a trio *via* a newly conceived coupled Bloom Filter, then corrects sequencing errors by using a statistical approach and extends kept *k*-mers into long sequences. These extended sequences are used as input for variants needed. Later, the obtained variants are comprehensively analyzed against existing databases to mine VSD-related mutations. Experiments show that our trio-based algorithm narrows down candidate coding genes and lncRNAs by about 10- and 5-folds comparing with single sequence-based approaches, respectively. Meanwhile, our algorithm is 10 times faster and 2 magnitudes memory-frugal compared with existing state-of-the-art approach. By applying our approach to a VSD trio, we fish out an unreported gene—CD80, a combination of two genes—MYBPC3 and TRDN and a lncRNA—NONHSAT096266.2, which are highly likely to be VSD-related.

## Introduction

Ventricular septal defect (VSD) is a major kind of congenital heart disease (CHD), constituting about 20% of all CHD cases (Spicer et al., 2014). By taking conservative treatment, mortality is around 90% to 95%, whereas *via* surgery, this rate reduces to 19% to 60% (Serpytis et al., 2015). Very often, diagnosis of a VSD patient is at its late stage due to the obvious communication obstacles in infants; this poses a need for early diagnosis, particularly through genetic variants.

Mining genetic variants and associating them with diseases is a hot topic, by which thousands of disease-associated variants have been identified (International HapMap Consortium et al., 2010; The 1000 Genomes Project Consortium et al., 2015). Obtaining these findings usually starts with a panel containing hundreds to thousands of patients diagnosed as having the same specific disease; later their genetic materials are extracted and sequenced. This is followed by disease-associated variants mining through a series of analytic procedures. Using this protocol, 89,251 single-nucleotide polymorphism (SNP) trait associations have been successfully pinpointed according to genome-wide association study (GWAS) catalog (MacArthur et al., 2017), including more than 400 CHD-related genes (Jin et al., 2017a). Although an association study is fruitful and promising, many issues weaken its applicability. First, panel-based association studies only identify common variants, and rare variants are overlooked due to low statistical significance. Thus, it requires large number of samples to be collected, i.e., hundreds, even thousands of cases. Second, almost all existing studies mine a one-to-one correspondence between genes and diseases rather than a many-to-many scheme, which is pretty challenging. Unfortunately, majority of diseases are caused by many mutations of genes. For instance, more than 400 genes have been discovered to be associated with CHD (Jin et al., 2017a), and more than 700 genes are involved in adult height (Wood et al., 2014), and even much more (Marouli et al., 2017). Third, it is costly to obtain the whole DNA (deoxyribonucleic acid) sequence of a sample. Although the ever increasing throughput and decreasing cost have made whole-genome sequencing possible for general research, it still costs a few hundreds to a thousand dollars for a single genome. To partially overcome the aforementioned limitations from single sequencing (SS) data, trio-based sequencing emerges.

Typically, a trio usually contains two parents and one child. This trio-based approach is effective for identifying disease-associated genes according to the basic rule of inheritance. It is also powerful to pinpoint *de novo* mutations without a large panel. Various studies have been conducted to identify disease-associated genes by using trio-sequencing (TS). For instance, a trio-based exome sequencing is used to identify *de novo* mutations in early-onset high myopia (Jin et al., 2017b), and ∼440 CHD-related genes have been discovered based on 2,645 trios (Jin et al., 2017a). The typical procedure of using trios to identify variants is mapping-calling-filtering, i.e., mapping all sequences of each individual from a trio to a reference genome, calling variants based on mapped sequences, and filtering out variants shared by members of the trio. Intuitively, this protocol is inefficient to identify *de novo* mutations from child sequences. Obviously, a large portion of sequences have no contribution to variant calling, which have been considered during the whole processes for all samples within the trio. To solve this problem, we propose a novel idea of calling *de novo* variants from a trio and have applied it to identified VSD-related genetic variants, including coding genes and long non-coding RNAs (lncRNAs).

Our approach starts from a trio with a child diagnosed as having VSD but with healthy parents. Later, unique *k*-mers (*k*-length consecutive bases from a genomic sequence) belonging to the child only are fished out through a newly proposed counted *k*-mer-encoding algorithm. This is followed by sequence error correction and *k*-mer extension before mapping to a reference genome. Finally, variants are fished out and analyzed against existing databases to mine VSD-related coding genes and lncRNAs.

## Methods

Our approach is composed of two major parts: TS-based variant mining and VSD-related variant filtering.

### Variant Mining

Unlike conventional mapping-calling-filtering approach of variant identification, e.g., SAMtools (Li et al., 2009) and GATK (DePristo et al., 2011), we conceive a novel idea of *de novo* variants identification algorithm from a trio achieving good computation efficiency. Our approach contains four steps: *k*-mer filtering, *k*-mer extension, and variant identification. Details are shown below.

#### *k*-mer Filtering

Let a trio be *R_f_*, *R_m_*, *R_c_*, representing the reads of the father (*R_f_*), the reads of the mother (*R_m_*), and the reads of the child (*R_c_*, suppose only one child is available); the set of *k*-mers contained in a sample is *K_f_*, *K_m_*, and *K_c_* for father, mother, and child, respectively. Herein, we mean each *k*-mer having its count (the times it appears within the sequenced data) available, i.e., a *k*-mer, say κ, is a touple (*s*_κ_, *f*_κ_), where *s*_κ_ is the *k*-length string of κ, and *f*_κ_ is its count. To fish out *de novo* mutations from *K_c_*, we go through all the *k*-mers of *K_c_* and check them with *K_f_* and *K_m_*. In case the count ratio of a *k*-mer between both parents and the child is less than a threshold (τ_0_), the *k*-mer is kept as a variant-containing candidate.

It seems trivial to filter out large amount of *k*-mers shared between *K_f_*/*K_m_* and *K_c_*. However, the number of *k*-mers obtained from a whole human genome sequencing reads is usually too large to fit into a main memory, not to mention putting them together. For instance, the 31-mers having a count larger than one of the HapMap sample NA12878 ((https://www.ncbi.nlm.nih.gov/sra/ERR091571/)) take 90-Gb space on disk. To solve this problem, we have designed a novel coupled Bloom Filter-based algorithm achieving high memory saving ratio and good retrieval efficiency (Jiang et al., 2019). Let *f*_max_ be the maximum frequency in *K*, which can be represented by at most *h* bits (in binary). We take the following steps to represent *K*:

1. Create a hash function vector *H* containing *h* hash functions, say 〈H_0_ (·), *H*_1_ (·), ⋯, *H_h_*_−1_(·)〉.
2. Allocate a coupled Bloom Filter *B* = (*B* ^+^, *B* ^–^) having *m* bits. *m* is computed as $m=-\frac{n\ln p}{{\left(\ln2\right)}^{2}}$ with the target false-positive rate *p* and number of *k*-mers *n* = |*K*|; *cf.* Bloom (1970).
3. For a *k*-mer κ in *K*, set the corresponding bits of *B* indexed by *H* as
  $$\begin{array}{ll} B^{+}\left[H_{i}\left(s_{\kappa }\right)\right]=1, & i\in \left(0,1,\cdots ,h-1\right),\\ B^{-}\left[H_{i}\left(s_{\kappa }\right)\right]=\text{Binary}\;{\left(f_{\kappa }\right)}^{h}\left[i\right], & i\in \left(0,1,\cdots ,h-1\right), \end{array}$$
  where binary (*f*_κ_)*^h^* is the binary representation of *f*_κ_ *via h* bits, and binary (*f*_κ_)*^h^*[*i*] returns the value of the *i*th bit.
4. Repeat Step 3 above until all *k*-mers are inserted.

Based on the above steps, *K_f_*, *K*_m_, and *K_c_* can be saved into *B*_f_, *B*_m_, and *B*_c_ economically; more details are shown in Jiang et al. (2019).

Based on above algorithm, we are able to store *K_f_*, *K*_m_, and *K*_c_ within a memory simultaneously and compute the ratio of a *k*-mer between a parent and the child efficiently. Note that, the time efficiency of *k*-mer retrieval from a coupled Bloom Filter mainly comes from the hash operation, which is in O(1) time complexity.

Due to sequencing bias, the *k*-mers are error-prone. To mitigate the impact of errors on variants identification, we perform error correction before further analysis (Zhao et al., 2018). For a *k*-mer κ in *K_x_*, we search its neighbors *N*_κ_ from *B_x_*, where *x* ∈{*f*, *m*, *c*}. A neighbor of κ is defined as the one having edit distance of 1 from κ. Later, a *z*-score *z*_κ_ is calculated from ${N^{\prime }}_{\kappa }=N_{\kappa }\cup \{\kappa \}$, where *z*_κ_ = (*f*_κ_ – μ)/σ, μ is mean frequency of *k*-mers in ${N^{\prime }}_{\kappa }$, and σ is their standard deviation. We consider κ is error-free when *z*_κ_ > *z*_0_ and *f*_κ_ > *f*_0_. In this study, *z*_0_ = 0.8 and *f*_0_ = 4. More details are presented in Zhao et al. (2018).

**Algorithm 1:** *k*-mer filtering.

**Data**: (*K_f_*, *K_m_*, *K_c_*), *k*-mers of a trio; *H*, a hash function vector **Result**: ${K^{\prime }}_{c}$, mutation-contained *k*-mers of a child **begin** **for** *x* in {*f*, *m*, *c*} **do** *B_x_* = Encoding(*K_x_*, *H*) **for** κ in *K_c_* **do** *ν_f_* ← Decoding(*B_f_*, *H*, *s*_κ_) *ν_m_* ← Decoding(*B_m_*, *H*, *s*_κ_) *ν_c_* ← Decoding(*B_c_*, *H*, *s*_κ_) **if** *u_f_*/*ν_c_* < τ_0_ **and** *u_m_*/*ν_c_* < τ_0_ **then** ${K^{\prime }}_{c}$ ← Correction(*B_c_*, *H*, ${K^{\prime }}_{c}$) **return** ${K^{\prime }}_{c}$ //Details of *Encoding*, *Decoding* and *Correction* are shown in Appendix A.

#### *k*-mer Extension

A *k*-mer is usually not long enough to uniquely map to a specific location of a reference genome. Hence, extending a *k*-mer into a long sequence is necessary before mapping. To this end, we take a candidate variation-containing *k*-mer as seed, and elongate the *k*-mer to both side. Taking right-hand extension, each time one base is attached to the right of the current string *s*, i.e., *s*ʹ = *s *⋅ *x*, *x* ∈{*A*, *C*, *G*, *T}*, and the *k* length suffix of *s*ʹ, i.e., $\text{suffix}{\left(s^{\prime }\right)}_{k}={s^{\prime }}_{\left[\left(l-k-1\right):l\right]}$, is checked against *B*_c_. In case the suffix is absent, the extension will be altered by another base, or terminated if all alternatives have failed. The left-hand side extension is similar to the right-hand extension but with opposite direction. An extension will be terminated in case the length limitation is reached or multiple extensions are available. We set the length limitation to 1,000 in this study. Extension details are shown in Algorithm 2.

**Algorithm 2:** *k*-mer extension.

**Data**: *B_c_*, child *k*-mers; *H*, a hash function vector; ${K^{\prime }}_{c}$, kept *k*-mers; maxLen: maximum length **Result**: *S*, set of variant-containing sequences **begin** **for** κ in ${K^{\prime }}_{c}$ **do** hasBranch ← 0 *s*′ ← *s*_κ_ **repeat** *c* ← 0, *e* ← ‘‘ **for** *x in* {*A, C, G, T*} **do** *s*″ ← suffix(*s*′, *k* – 1) · *x* val ← Decoding(*B_c_*, *H*, *s*″) **if** val > *0* **then** *c* ← *c* + 1, *e* ← *x* **if** *c* > 1 **then** hasBranch ← 1 **else** *s*′ ← *s*′ · *e* **until** *hasBranch* **or** |*s*′| > *maxLen* hasBranch ← 0 **repeat** *c* ← 0, *e* ← ‘‘ **for** *x in* {*A, C, G, T*} **do** *s*″ ← *x* · prefix(*s*′, *k* – 1) val ← Decoding(*B_c_*, *H*, *s*″) **if** val > *0* **then** *c* ← *c* + 1, *e* ← *x* **if** *c* > 1 **then** hasBranch ← 1 **else** *s*′ ← *e* · *s*′ **until** *hasBranch* **or** |*s*′| > maxLen $\text{S}\leftarrow \text{S}\cup \{s^{\prime }\}$ **return** *S*

#### Variant Identification

All extended *k*-mers are mapped to GRCh38/hg38 by BWA (Li and Durbin, 2009), and variants as well as their position are pinpointed by using SAMtools (Li et al., 2009) and the best practice of GATK (DePristo et al., 2011). Unlike most existing approach that uses read coverage to filter out low confidence variants, we use previously identified variant-containing *k*-mers (from the first step and with count included) to refine the obtained variants. More precisely, a variant is kept if it satisfies the following criteria: 1) a *k*-mer (formed by the reference genome and the variant jointly) containing the variant can be found from the set of kept *k*-mers obtained from the first step; 2) the sum of the count of all *k*-mer covering the variant is not less than 3. Note that the first criterion is necessary because extended *k*-mers introduce additional variants that are not unique to the child.

### Variant Filtering

We focus on VSD-related variants; thus, those obtained from the previous step undergo filtering to fish out VSD-related variants. Two types of variants are considered, viz., contained in coding and non-coding regions. For affected non-coding genes, we pay special attention on long non-coding RNAs.

#### Identifying VSD-Related Variants

Only a tiny portion of variants obtained from GATK could be VSD-related. To obtain these variants, we first filter out irrelevant ones by GATK built-in modules with various parameters, including “QD < 2.0,” “QUAL < 30.0,” “SOR > 3.0,” “FS > 60.0,” “MQ < 40.0,” “MQRankSum<-12.5,” and “ReadPosRankSum<-8.0” for SNPs and “QD < 2.0,” “QUAL < 30.0,” “FS > 200.0,” and “ReadPosRankSum<-20.0” for indels (insertions and deletions). This step is followed by using ANNOVAR (Wang et al., 2010) to filter out variants presented in known individuals with minor allele frequency (MAF) of 0.01. Reference databases used in this stage are the phase 3 of 1000 Genomes Project (The 1000 Genomes Project Consortium et al., 2015), ExAC (Lek et al., 2016), ESP (Exome Variant Server, 2019), and gnomAD (Lek et al., 2016). That is, a variant that appears in these databases having MAF no less than 0.01 is excluded.

After filtering, we use DAVID (Huang et al., 2009) to analyze functions of remaining variants. These variants are also validated by using Gene Ontology (GO) (The Gene Ontology Consortium, 2017), Kyoto Encyclopedia of Genes and Genomes (KEGG) (Kanehisa et al., 2018), the Online Mendelian Inheritance in Man (OMIM) (Hamosh et al., 2005), and the Human Gene Mutation Database (HGMD) (Stenson et al., 2017). Functions and pathways of coding genes can be easily obtained by using DAVID, whereas they are unable to be obtained directly for non-coding transcripts. Hence, we handle them separately; see below.

#### Fishing Out Coding Genes

Taking the results generated by ANNOVAR, we select the variants having consequence of “Nonsense_Mutation,” “Frame_Shift_Ins,” “Frame_Shift_Del,” “Translation_Start_Site,” “Splice_Site,” “In_Frame_Ins,” “In_Frame_Del,” and “Missense_Mutation.” In addition, variants having SIFT score (Sim et al., 2012) larger than 0.05 and PolyPhen-2 index (Adzhubei et al., 2010) smaller than 0.446 are further filtered out. The remaining genes are input into DAVID to analyze gene-disease association, gene-annotation enrichment analysis, pathway mapping, and so on. Those genes related to cardiovascular diseases are fished out. In addition, the neurodegenerative diseases-related genes are also obtained because many studies have shown that these two diseases are closely related (Jin et al., 2017a).

The pinpointed genes are also checked with GO, OMIM, HGMD, and KEGG to verify their functions if available.

#### Pinpointing Out lncRNAs

A lncRNA does not translate proteins; however, it possesses many roles in gene transcription regulation, post-transcriptional regulation, epigenetic regulation, aging, and so on (Marchese et al., 2017). Hence, mutations occurred in lncRNAs may affect the downstream products. To identify the VSD-related variant-containing lncRNAs within the child, we extend the VSD-related genes (listed in Jin et al., 2017a) to upstream and downstream by 100, 200, 500, and 1,000 bp. A variant is considered as a candidate if it is within the extension region and overlaps with lncRNAs shown in LNCipedia (Volders et al., 2018) or NONCODE (Zhao et al., 2016). Note that this protocol approaches the VSD-related lncRNAs approximately but not directly. The rationale is that regulatory elements within proximity usually play a role together (Razin et al., 2013; Andrey and Mundlos, 2017).

Functions of identified lncRNAs are fully explored by using LNCipedia and NONCODE.

## Results

### Data Preparation

A trio containing a 3-year-old boy diagnosed as having typical VSD and a couple of healthy parents is collected. The DNA of each individual is extracted from 5-ml venous blood and is sequenced by an Illumina HiSeq X Ten platform having coverage of 30× and read length of 151 bp. All DNA sequences of the three samples are obtained from one batch. As a result, 356,781,358 paired-end reads are obtained from the child, and 368,280,232 and 330,790,178 paired-end reads are obtained from his father and mother, respectively.

Before the sample collection, a written informed consent is obtained from the parents of the child.

### TS-Based Variants

Based on the TS data, we obtained 2,585,348 variants by using GATK (DePristo et al., 2011) with default settings. These variants are further divided into two types, i.e., protein coding and non-coding. For the non-coding variants, we focus on the regions transcribed into lncRNAs. Variants associated with both cardiovascular and neurodegenerative diseases are explored because they usually occur together (Jin et al., 2017a). Details are shown below.

#### Coding Genes Related to VSD

From the 2,585,348 variants, 193 within exonic regions and 6 from splicing regions pass various filtering criteria that are obtained by using ANNOVAR (Wang et al., 2010). The 193 variants are associated with 61 unique genes, whereas the 6 are involved in 8 genes; see more details in the **Supplementary File**.

**Figure 1:**
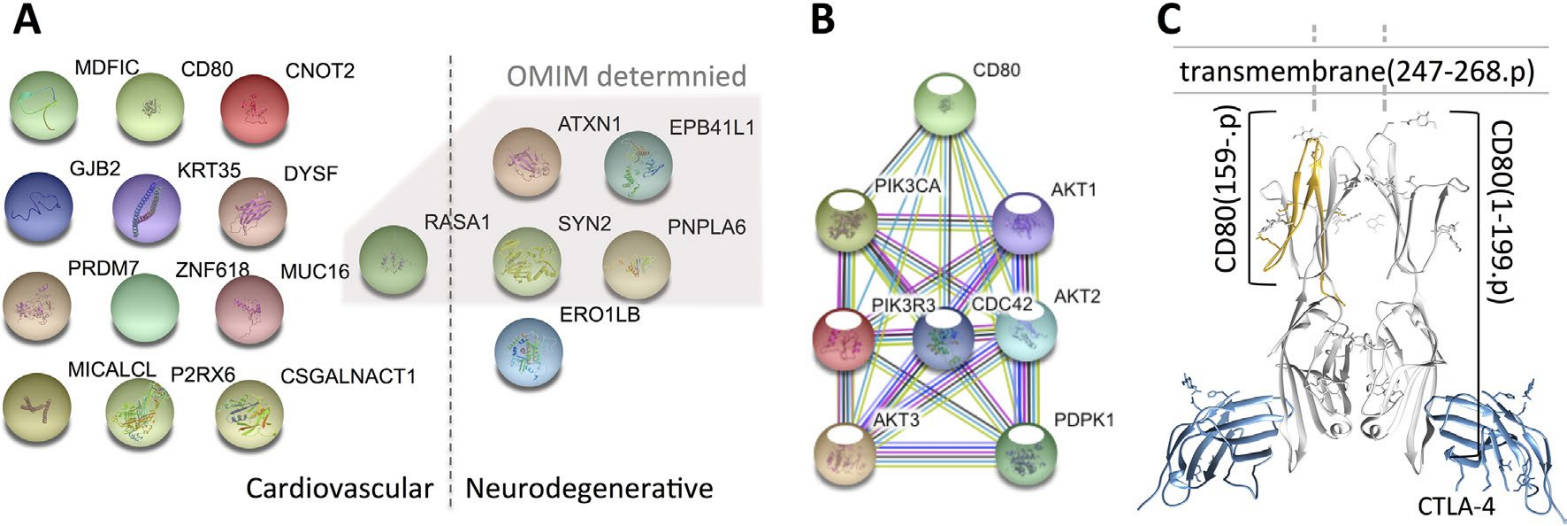
Variant-containing coding genes obtained from the trio that are associated with cardiovascular and neurodegenerative diseases. Panel **(A)** shows the 18 genes attached to the two categories [generated by using the STRING database (Szklarczyk et al., 2017)], panel **(B)** presents the connections between CD80- and CHD-related genes, and panel **(C)** illustrates the 3D structure of the mutated CD80 (PDB ID: 1I8L) discovered in this study. The 18 genes are fished out by using DAVID from OMIM and GAD databases, genes identified by OMIM are shaded by a polygon. Note that, all the genes identified by OMIM have also been confirmed by GAD.

Taking the 61 genes as input, we identify 14 genes related to cardiovascular diseases, including RASA1, CNOT2, MICALCL, MDFIC, PRDM7, ATXN1, CSGALNACT1, DYSF, GJB2, KRT35, MUC16, P2RX6, ZNF618, and CD80, and 5 genes related to neurodegenerative diseases, which are ATXN1, EPB41L1, PNPLA6, SYN2, and ERO1B (see **Figure 1** and **Table 1**). Among the 18 genes (ATXN1 appears in both categories), 5 of them have been confirmed by OMIM and GAD (Genetic Association Database) (Becker et al., 2004), of which 2 (RASA1 and ATXN1) are cardiovascular disease-related and 4 are neurodegenerative disease-related (ATXN1, EPB41L1, PNPLA6, SYN2), whereas the rest only appear in one database. Compared with all the 457 VSD-related genes shown in Jin et al. (2017a), we found that MUC16 is common for both data.

**Table 1:** Details of the 16 variant-containing coding genes identified from the trio.

Disease	Gene	Chr.	Pos.	Reference	Alt.	Variant^a^	|Transcript|^b^	Coverage^c^	MAF*^d^*
	DYSF	2	71665193	C	T	MM	14	36	0.52
	MUC16	19	8888863	T	C	MM	4	55	0.52
	P2RX6	22	21023596	C	T	NM	4	33	0.54
	ZNF618	9	114050108	C	T	MM	4	35	0.57
	CD80	3	119537362	T	—	FSD	3	39	0.56
	CNOT2	12	70353914	A	—	IFD	3	15	0.40
Cardiovascular	ATXN1	6	16327634	TGCTGC	—	IFD	2	16	0.31
	RASA1	5	87383769	A	G	MM	2	21	0.57
	MDFIC	7	114922989	C	G	MM	2	18	0.66
	CSGALNACT1	8	19458429	A	T	MM	2	27	0.62
	PRDM7	16	90058384	G	—	FSD	1	37	0.64
	MICALCL	11	12294797	—	CTCCTC	IFI	1	11	0.36
	GJB2	13	20189347	G	—	FSD	1	33	0.39
	KRT35	17	41477614	C	T	MM	1	31	0.41
Neurodegenerative	PNPLA6	19	7556658	C	A	MM	5	26	0.46
	EPB41L1	20	36209768	C	T	MM	4	35	0.62
	SYN2	3	12004751	—	GCCCGCGCCGCA	IFI	2	6	0.33
	ATXN1	6	16327634	TGCTGC	—	IFD	2	20	0.31
	ERO1B	1	236235819	G	A	MM	1	21	0.52

^a^Variant classes include MM (missense mutation), NM (nonsense mutation), FSD (frame shift deletion), IFD (in frame deletion), IFI (in frame insertion), IFD (in frame deletion); ^b^affected number of transcripts, ^c^reads coverage, and ^d^mutant allele frequency.

We also performed pathway enrichment test for the identified genes; however, no significant cardiovascular-related pathway can be identified. We found that only five genes overlap with the genes saved in KEGG.

After careful investigation of the 14 cardiovascular disease-related genes *via* literature review, we found 13 of them have literature support that are associated with CHD (VSD is the most common type of CHD), while CD80 has no explicit support. Hence, we take further effort to explore the possible roles of CD80 in VSD development.

CD80 is well known in providing co-stimulatory signal necessary for T-cell activation and survival, which has been found on dendritic cells, activated B cells, and monocytes (Peach et al., 1995). To our knowledge, no study shows direct relation between CD80 and CHD. However, Kallikourdis et al. (2017) have reported that CD80 involved in T-cell costimulation complex contributes to a heart failure, suggesting that mutated CD80 has impact on heart defect. Hence, we carefully explored the role of CD80 with VSD computationally.

To examine whether the mutated CD80 has a connection with VSD as shown in this study, we retrieved all cardiovascular-related genes from GO, OMIM, and HGMD, and built connections between these genes and CD80 by using the STRING database (Szklarczyk et al., 2017). Results show that 31 genes in GO and 18 genes in OMIM have connections with CD80 (in protein association, including known interactions, predicted interactions, co-expression, etc.). In total, 41 genes have connection with CD80. The details are shown in **Table 2**. Among these 41 genes, 7 of them are known interactions (experimentally determined or curated from databases, shown in italic in **Table 2**; see **Figure 1B**). Among these genes, AKT1, PDPK1, CDC42, AKT3, and PIK3CA have concrete evidences shown in relation with congenital heart disease; even two of them (AKT1, CDC42) have explicit association with VSD (Chang et al., 2010; Liu et al., 2017).

**Table 2:** CD80 interacting genes in GO and OMIM that are associated with cardiovascular diseases.

	*AKT1*	*PDPK1*	*CDC42*	*PIK3R3*	*AKT3*	*PIK3CA*	PIK3CB	TGFB1
CD80-GO	PTEN	IL8	CXCL10	IL10	CCL2	CCR2	THY1	ERBB2
PIK3CG	TLR3	IL1B	CD34	ANPEP	STAT3	FASLG	VEGFA
JUN	IL18	NRP1	IL6	STAT1	CD40	CXCR3	
CD80-OMIM	AKT2	ICAM1	ITIH4	PIK3CG	CD36	CD40LG	NRP1	IL10
	IL6	CD40	SCARB1	INS	IFNA1	LMNA	IL4	VEGFA
	IL18	PTEN						

Genes in italic are experimentally determined that have interactions with CD80, whereas the rest are computationally predicted.

Regarding the mutated CD80 in this study, it has a “T” deletion on the reverse strand at chr3:119537362, leading to a frame shift at position 159 of the translated protein (cf. **Figure 1C**). As a result, the protein is no longer able to insert into the membrane of a cardiac myocyte; see **Figure 1C**. Therefore, the downstream pathway will be affected.

Regarding the eight variants that reside in the splicing region, we found only one (MROH5) that is related to cardiovascular disease.

#### LncRNAs Related to VSD

Other than fishing out candidate VSD-related genes from variants directly, we use known VSD-associated genes as a seed, and then pinpoint lncRNAs having variants near these seeds. A set of 457 known VSD-related genes are obtained from Jin et al. (2017a), whereas the whole set of lncRNAs are retrieved from LNCipedia and NONCODE. A variant-containing lncRNA is considered to be VSD-related if it is within a certain distance of a known VSD-related gene. Other than using a single distance, we use various distances, which are 100, 200, 500, and 1,000 bp.

We identified 6, 7, 27, and 49 lncRNAs from LNCipedia having distance of 100, 200, 500, and 1,000 bp, respectively, whereas these numbers are 6, 8, 32, and 57 when checked against NONCODE. Details are shown in the **Supplementary File**. To examine whether these lncRNAs have a potential effect to VSD, we carefully studied their expression in different tissues, particularly in the heart. We found that among all the lncRNAs (both from LNCipedia and NONCODE), 29 of them present in the heart, especially NONHSAT096266.2 (NONCODE ID), which is highly and uniquely expressed in the heart, having a FPKM score of 13.97. More interestingly, this lncRNA is very close to NFXL1, which has been identified as a VSD-associated gene (Jin et al., 2017a). See **Table 3**.

**Table 3:** The top 10 lncRNAs obtained from the trio that have potential relation with VSD.

NONCODE ID	Chr.	Pos.	Ref.	Alt.	FPKM	Dis.(bp)
NONHSAT096266.2	4	47846448	C	T	13.97	1000
NONHSAT232531.1	12	131923913	G	A	2.08	1000
NONHSAT001273.2	1	19074132	T	C	1.99	1000
NONHSAT180457.1	19	44259481	T	C	1.54	1000
NONHSAT235401.1	15	66701900	G	A	1.49	1000
NONHSAT244710.1	22	19179167	A	G	1.15	1000
NONHSAT010771.2	1	247332298	AC	A	0.89	1000
NONHSAT022678.2	11	71452973	T	C	0.37	1000
NONHSAT229850.1	11	64779243	GAAAAAA	G	0.32	1000
NONHSAT246250.1	3	9396926	G	A	0.22	1000

FPKM: fragments per kilobase of exon per million reads mapped (Mortazavi et al., 2008).

#### Results Comparison

We use TrioDeNovo (Wei et al., 2015) with default settings (depth of coverage equals 5) to call *de novo* variants from the trio and compare results with that of our approach. As a result, TrioDeNovo identifies 79,082 variants contained in 357 genes. After filtering out common variants with MAF of 0.01, 51 variants located in 25 genes are obtained. Among these genes, 21 overlap with our findings. Regarding lncRNAs, no variant can be found within 1,000 bp of known VSD-related genes.

### SS-Based Variants

Intuitively a TS-based approach is able to significantly narrow down candidate genes; however, it is hard to speculate to what extent the improvement is. Hence, we have conducted experiments on the sequences of the VSD sample (the child) only with the same protocols as the TS-based experiments.

Based on the single-sequencing (SS) data, we have obtained 4,826,899 variants by using GATK (DePristo et al., 2011). Similar as trio-sequencing (TS) data analysis, we divide them into protein coding and non-coding variants.

#### Coding Genes Related to VSD

After annotation and filtering by using ANNOVAR, we obtained 1,552 variants contained in 436 genes. Among these genes, 424 have exonic variations and the other 12 have splicing variations. For the 424 genes, we identified MYBPC3 (Chr11:47342683, C/T, missense mutation, p.G507R) and TRDN (Chr6:123571021, C/A, missense mutation, p.S45I), which are highly related to ventricles, by using DAVID based on OMIM. More details are shown in the **Supplementary File**. Unfortunately, these two genes cannot be identified based on the trio. Further investigation has shown that the variation in MYBPC3 is inherited from the father, whereas the variation in TRDN is inherited from the mother. Considering the truth that both the father and mother are healthy, but the child has VSD, we speculate that the combination of mutated MYBPC3 and TRDN may have noteworthy contribution to VSD. Regarding the 12 genes having variants in splicing regions, we have not found cardiovascular- or neurodegenerative-related genes.

The genes identified by GAD are excluded for the child sample analysis. This is because more than 60 genes can be found, and the significance of relations between these variants and VSD can be hardly determined.

#### LncRNAs Related to VSD

Similar to TS-based lncRNA identification, we carried out the same experiments on the VSD patient only. Unlike the results obtained from coding genes that are about 10 times larger, candidates are selected from the SS-based data than the TS-based data; we get five times larger number of lncRNA variants between the SS-based data and the TS-based data.

The numbers of lncRNAs having variants close to VSD-related genes are 37, 60, 129, and 197 for distances of 100, 200, 500, and 1,000 bp, respectively. Among all these lncRNAs, only 97 are present in heart cells, of which 23 are highly expressed having FPKM score larger than 1. Details are shown in the **Supplementary File**. For instance, the lncRNA NONHSAT181468.1 (Chr2:27217145, CT/C), which has the highest FPKM score of the identified lncRNAs, is highly expressed in the heart having FPKM of 31.7. This lncRNA is within the first intron of SLC5A6, which has been confirmed as a VSD-associated gene.

### Variants Profile of the Trio

**Figure 2:**
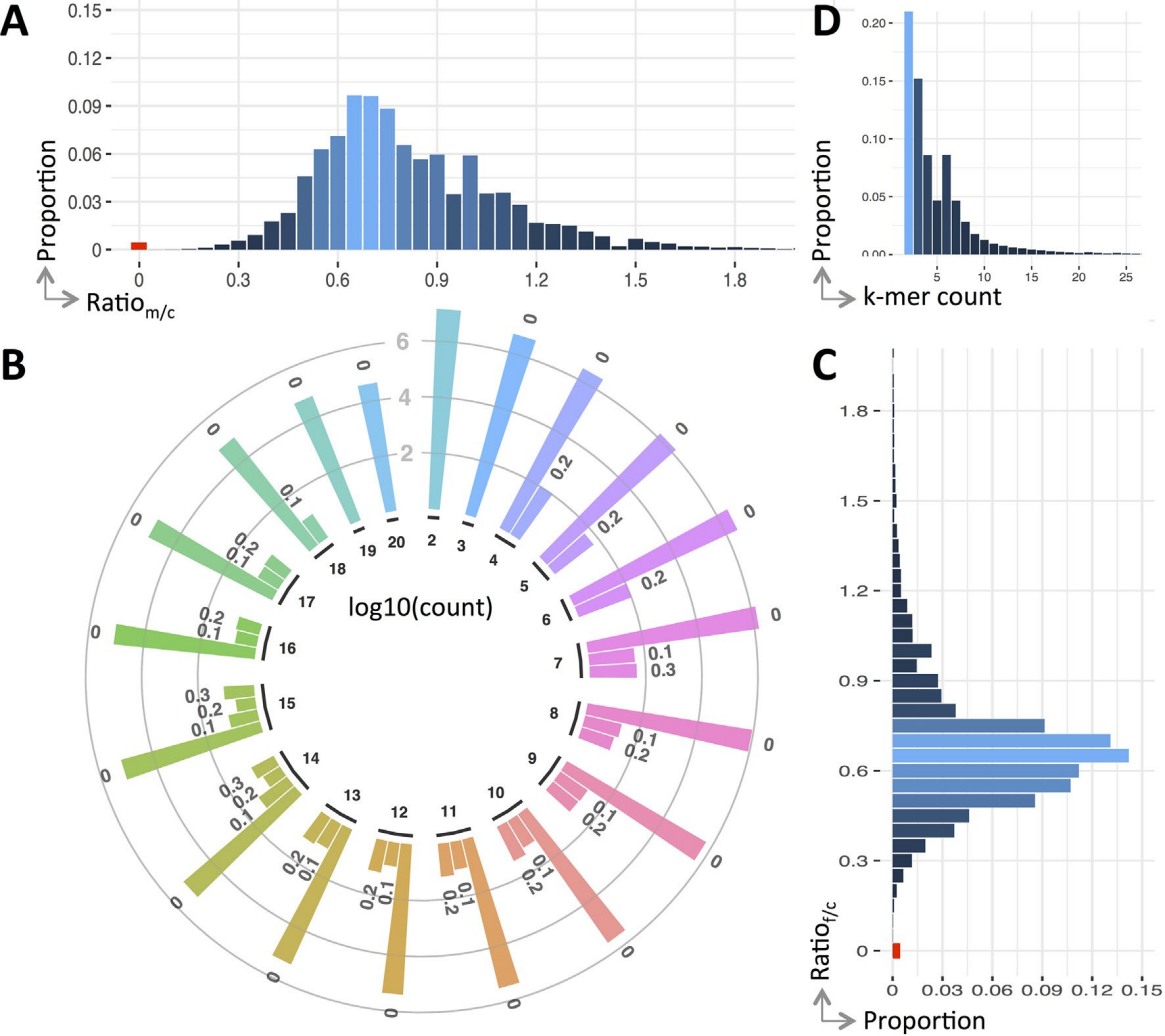
Variants profile of the trio. Panels **(A** and **C)** are the distribution of *k*-mer count ratio between the parents and the child, panel **(B)** is the ratio distribution of the *k*-mers having a count less than 20, and panel **(D)** is the overall distribution of ratio against count. Note that ratio distribution in panel **(B)** is clustered into bins, *viz*., *r* = 0 (denoted as “0”), 0 < r ≤ 0.1 (denoted as “0.1”), 0.1 < *r* ≤ 0.2 (denoted as “0.2”), and 0.2 < *r* ≤ 0.3 (denoted as “0.3”), where *r* is a ratio and the count is in log scale.

We use the ratio of *k*-mers between the parents and child to reflect their genetic variations. **Figure 2** shows the detailed ratio distribution. It is clear that only a small portion of *k*-mers have ratio of 0 (see **Figure 2A, C**). That having been said, among the *k*-mers of the child, only 0.442% contains *de novo* mutations compared with his father, and this value is 0.438% compared with his mother. After combining them together, 0.43% are unique *k*-mers.

The ratio of *k*-mers may be affected by sequencing errors. To alleviate this impact, we include *k*-mers having small ratio (less than 0.3) except the ones having a ratio of 0. Generally, the number of *k*-mers having a ratio of 0 is four to five magnitudes larger than those non-zero ones (see **Figure 2C**). In case these *k*-mers contain mutations, they will be fished out during the downstream variant calling.

Unlike the distribution of *k*-mer count for all k-mers (approximate normal), the *k*-mers having mutations follow a Poisson distribution (see **Figure 2D**). The *k*-mers having counted smaller than 20 forms 97.97% of all *k*-mers having ratio less than 0.3. The distribution breakdowns of these *k*-mers are shown in **Figure 2B**.

### Run-Time Analysis

Our experiments are conducted on a computer having 128G RAM and two E5-2683V4 CPUs (32 cores in total), installed with CentOS 7.0. Throughout the entire experiments, we use 24 threads as default if applicable.

Other than existing approaches that filter out irrelevant variants from trios after mapping, e.g., TrioDeNovo (Wei et al., 2015), we conduct filtering before mapping. This small change is not trivial since the input data are usually very large. For instance, the input size of the VSD sample used in this study is 242 Gb in fastq format, and the total size is over 700 Gb for the trio. To solve this problem, we have conceived a novel coupled Bloom Filter-based *k*-mer encoding algorithm. This algorithm achieves a compression ratio of 12 under default settings. That having been said, a typical set of *k*-mers obtained from a human genome (usually around 120Gb) can be compressed into 10 Gb. Using this approach, we are able to handle a trio within a main memory.

Experiments show that the total memory used to encode counted *k*-mers obtained from the trio is 31.7 Gb. Based on the encoded *k*-mers having count available, we calculate count ratio of all *k*-mers between the parents and the child. Mathematically, suppose the count of a *k*-mer κ from the child is $f_{c}^{\kappa }$, and the count is $f_{f}^{\kappa }$ and $f_{m}^{\kappa }$ for his father and mother, respectively; then, the count ratio is $r_{f/c}^{\kappa }=f_{f}^{\kappa }/f_{c}^{\kappa }$ between his father and himself. Analogously, the count ratio between his mother and himself is $r_{m/c}^{\kappa }=f_{m}^{\kappa }f_{c}^{\kappa }$. If both $r_{f/c}^{\kappa }$ and $r_{m/c}^{\kappa }$ are smaller than the threshold *r*_0_, then, κ is kept, where *r*_0_ is set as 0.3 in this study. Results show that *k*-mer counting takes 129 min, *k*-mer encoding takes 175 min, and *k*-mer filtering takes 20.3 s. As a result, 3.9% *k*-mers are left for further analysis.

Because there exist sequencing errors, we perform error correction on the remained *k*-mers (Zhao et al., 2017). It takes 1.7 s and 0.12-Gb RAM to correct 93.7% errors of the kept *k*-mers. As a result, 293.2M *k*-mers are left for variants identification.

Before mapping variant-containing *k*-mers to a reference genome, we have also conducted *k*-mer extension to avoid multi-mapping problem caused by short input sequence, e.g., *k*-mer. An extension takes a *k*-mer as seed, and extends the *k*-mer to both sides based on the reads in which the *k*-mer is contained. Finally, we mapped extended sequences to the reference genome GRCh38/h38 *via* BWA (Li and Durbin, 2009), which takes 52 min to finish. This is followed by variants calling through SAMtools Li et al. (2009) and GATK (DePristo et al., 2011) jointly. It takes 50 min to finish the above mentioned steps.

Regarding TrioDeNovo, it takes 572 min to get the sorted sam file from a raw fastq file and uses 8,179 min to merge and generate the final vcf file by using GATK and TrioDeNovo. Compared with our approach, TrioDenovo is 10 times (= (8179 + 572*3)/((129 + 175)*3+102)) slower than ours. Besides, the maximum RAM required by our approach is two magnitudes smaller.

## Conclusions

As the most common CHD, VSD affects a noteworthy portion of newborns, leading to a high mortality. Unveiling the biological mechanism, particularly the underpinning genetic variants, is essential for both early diagnosis and clinical treatment. Existing approaches of mining genetic variants rely on large panels, which is challenging in cost and sample collection. It is also prone to overlooking rare variants and hard to handle multiple variants. We designed a novel algorithm for identifying variants from a trio and associate them with VSD. Experiments show that trio-sequencing-based approach is able to narrow down VSD-related candidates by about 10 times in coding genes and 5 times in lncRNAs; meanwhile our approach is 10 times faster than existing state-of-the-art approach. Applying our method to a VSD trio, we fish out 14 coding genes closely correlated to cardiovascular diseases and 5 coding genes associated with neurodegenerative diseases. Among them, CD80 has not been reported yet. More promisingly, results show that the combination of MYBPC3 and TRDN has high possibility to be VSD-related. Analysis on lncRNA shows that six are highly expressed in heart that are within 1,000 bp to VSD-related genes, particularly NONHSAT096266.2, which has a FPKM socre of 13.97 and is uniquely expressed in heart.

## Author Contributions

LZ conceived the algorithm, designed the experiments, and wrote the manuscript. PJ participated in program coding. PJ, YH, YW, JZ, LB, QT, and TL participated in data analysis. All authors read and approved the final manuscript.

## Funding

This study is collectively supported by the Free Exploration Fund of Hubei University of Medicine (FDFR201805), the National Natural Science Foundation of China (31501070), the Natural Science Foundation of Hubei (2017CFB137) and Guangxi (2016GXNSFCA380006, 2018GXNSFAA281275 and 2018GXNSFAA138085), the Scientific Research Fund of GuangXi University (XGZ150316), and Taihe Hospital (2016JZ11).

## Conflict of Interest Statement

The authors declare that the research was conducted in the absence of any commercial or financial relationships that could be construed as a potential conflict of interest.

## Appendix A

The procedure of *Encoding*, *Decodin* *g* and error *Correction* are shown below.

**Table:**

**Function** *Encoding* (*K*, *H*): B = Ø, *f_max_* ← max (*F*(*K*)), *h* ← |Binary (*f_max_*)| **while** *K ≠ *Ø **do** initialize new *B^+^*, *B^–^* and *K*ʹ **for** κ in *K* **do** flag ← False, freq ← Binary(*f*_κ_) ***** Roll-back point **for** *i* ← 0 to *h* – 1 **do** j ← H_i_(*s*_κ_) **if** *B^+^*[ *j* ] = = 1 **and** *B* ^–^[ *j* ] ≠ freq[i] **then** flag ← True *K*ʹ ← *K*ʹ ∪{κ} **else** *B* ^+^ [ *j* ] ← 1, *B* ^–^ [ *j* ] ← freq[i] **If** *flag* = = *True* **then** roll back *B* to the point * break; *B* ← *B* ∪ {(*B* ^+^, *B* ^–^)}, *K* ← *K*ʹ **return** *B* **Function** *Decoding* *(B, H, s_κ_* *)* **:** *h* ← |*H*| **for** *i* ← 0 to *h* – 1 **do** **if** *B* ^+^[*H_i_*(*s_κ_*)] = = *0* **then** **return** False **for** *i* ← 0 to *h* – 1 **do** *b_i_* ← *B* ^–^ [*H_i_*(*s_κ_*)] val ← Denary (*b*_0_ *b*_1_⋯*b*_(_ *_h_*_–1)_) **return** val **Function** Correction (B, H, Kʹ)***:*** **For** κ in *K*ʹ **do** *u*_κ_ ← Decoding (*B*, *H,* κ) *N*κ ← {*ν*_κ_} **for** *i* ← 1 to *k* **do** **for** *x* in {*A, C, G, T*} **do** **if** *s*_κ_[*i*] ≠ *x* then *s*_κʹ_ ← *s*_κ [1:(_ *_i_*_ – 1)]_·*x*·*s*_κ[(_ *_i_*_ + 1): _ *_k_*_]_ *ν*_κʹ_ ← Decoding (*B*, *H*, *s*_κʹ_) *z*_κ_ ← (*ν*_κ_ – mean (*N*_κ_))/std(*N*_κ_) **if** not *z*_κ_ > *z*_0_ and ν_κ_ < *f*_0_ **then** ${K^{\prime }}_{c}\leftarrow {K^{\prime }}_{c}-\kappa$ **return** ${K^{\prime }}_{c}$

## References

[B1] Adzhubei I. A. Schmidt S. Peshkin L. Ramensky V. E. Gerasimova A. Bork P. (2010). A method and server for predicting damaging missense mutations. Nat. Methods 7, 248–249. 10.1038/nmeth0410-248 20354512 PMC2855889

[B2] Andrey G. Mundlos S. (2017). The three-dimensional genome: regulating gene expression during pluripotency and development. Development 144, 3646–3658. 10.1242/dev.148304 29042476

[B3] Becker K. G. Barnes K. C. Bright T. J. Wang S. A. (2004). The genetic association database. Nat. Genet. 36, 431–432. 10.1038/ng0504-431 15118671

[B4] Bloom B. H. (1970). Space/time trade-offs in hash coding with allowable errors. Commun. ACM 13, 422–426. 10.1145/362686.362692

[B5] Chang Z. Zhang Q. Feng Q. Xu J. Teng T. Luan Q. (2010). Deletion of Akt1 causes heart defects and abnormal cardiomyocyte proliferation. Dev. Biol. 347, 384–391. 10.1016/j.ydbio.2010.08.033 20816796

[B6] DePristo M. Banks E. Poplin R. Garimella K. Maguire J. Hartl C. (2011). A framework for variation discovery and genotyping using next-generation DNA sequencing data. Nat. Genet. 43, 491–498. 10.1038/ng.806 21478889 PMC3083463

[B7] Exome Variant Server (2019). NHLBI GO Exome Sequencing Project (ESP). http://evs.gs.washington.edu/EVS/, Seattle, WA.

[B8] Hamosh A. Scott A. F. Amberger J. S. Bocchini C. A. McKusick V. A. (2005). Online mendelian inheritance in man (OMIM), a knowledgebase of human genes and genetic disorders. Nucleic Acids Res. 33, D514–D517. 10.1093/nar/gki033 15608251 PMC539987

[B9] Huang D. W. Sherman B. T. Lempicki R. A. (2009). Systematic and integrative analysis of large gene lists using DAVID bioinformatics resources. Nat. Protoc. 4, 44–57. 10.1038/nprot.2008.211 19131956

[B10] The International HapMap 3 Consortium (2010). Integrating common and rare genetic variation in diverse human populations. Nature 467, 52–58. 10.1038/nature09298 20811451 PMC3173859

[B11] Jiang P. Luo J. Wang Y. Deng P. Schmidt B. Tang X. (2019). kmcEx: memory-frugal and retrieval-efficient encoding of counted k-mers. Bioinformatics. 10.1093/bioinformatics/btz299 31038666

[B12] Jin S. C. Homsy J. Zaidi S. Lu Q. Morton S. DePalma S. R. (2017a). Contribution of rare inherited and *de novo* variants in 2,871 congenital heart disease probands. Nat. Genet. 49, 1593. 10.1038/ng.3970 28991257 PMC5675000

[B13] Jin Z.-B. Wu J. Huang X.-F. Feng C.-Y. Cai X.-B. Mao J.-Y. (2017b). Trio-based exome sequencing arrests *de novo* mutations in early-onset high myopia. Proc. Natl. Acad. Sci. U.S.A. 114, 4219–4224. 10.1073/pnas.1615970114 28373534 PMC5402409

[B14] Kallikourdis M. Martini E. Carullo P. Sardi C. Roselli G. Greco C. M. (2017). T cell costimulation blockade blunts pressure overload-induced heart failure. Nat. Commun. 8, 14680. 10.1038/ncomms14680 28262700 PMC5343521

[B15] Kanehisa M. Sato Y. Furumichi M. Morishima K. Tanabe M. (2018). New approach for understanding genome variations in KEGG. Nucleic Acids Res 47 (D1), D590–D595. 10.1093/nar/gky962 PMC6324070 30321428

[B16] Lek M. Karczewski K. J. Minikel E. V. Samocha K, E. Banks E. Fennell T. (2016). Analysis of protein-coding genetic variation in 60,706 humans. Nature 536, 285–291. 10.1038/nature19057 27535533 PMC5018207

[B17] Li H. Durbin R. (2009). Fast and accurate short read alignment with Burrows-Wheeler transform. Bioinformatics 25, 1754–1760. 10.1093/bioinformatics/btp324 19451168 PMC2705234

[B18] Li H. Handsaker B. Wysoker A. Fennell T. Ruan J. Homer N. (2009). The sequence alignment/map format and SAMtools. Bioinformatics 25, 2078–2079. 10.1093/bioinformatics/btp352 19505943 PMC2723002

[B19] Liu Y. Wang J. Li J. Wang R. Tharakan B. Zhang S. L. (2017). Deletion of Cdc42 in embryonic cardiomyocytes results in right ventricle hypoplasia. Clin. Transl. Med. 6, 40. 10.1186/s40169-017-0171-4 29101495 PMC5670094

[B20] MacArthur J. Bowler E. Cerezo M. Gil L. Hall P. Hastings E. (2017). The new NHGRI-EBI Catalog of published genome-wide association studies (GWAS Catalog). Nucleic Acids Res. 45, D896–D901. 10.1093/nar/gkw1133 27899670 PMC5210590

[B21] Marchese F. P. Raimondi I. Huarte M. (2017). The multidimensional mechanisms of long noncoding RNA function. Genome Biol. 18, 206. 10.1186/s13059-017-1348-2 29084573 PMC5663108

[B22] Marouli E. Graff M. Medina-Gomez C. Lo K. S. Wood A. R. Kjaer T. R. (2017). Rare and low-frequency coding variants alter human adult height. Nature 542, 186–190. 10.1038/nature21039 28146470 PMC5302847

[B23] Mortazavi A. Williams B. A. McCue K. Schaeffer L. Wold B. (2008). Mapping and quantifying mammalian transcriptomes by RNA-Seq. Nat. Methods 5, 621–628. 10.1038/nmeth.1226 18516045 PMC13303166

[B24] Peach R. J. Bajorath J. Naemura J. Leytze G. Greene J. Aruffo A. (1995). Both extracellular immunoglobin-like domains of CD80 contain residues critical for binding T cell surface receptors CTLA-4 and CD28. J. Biol. Chem. 270, 21181–21187. 10.1074/jbc.270.36.21181 7545666

[B25] Razin S. V. Gavrilov A. A. Loudinkova E. S. Larovaia O. V. (2013). Communication of genome regulatory elements in a folded chromosome. FEBS Lett. 587, 1840–1847. 10.1016/j.febslet.2013.04.027 23651551

[B26] Serpytis P. Karvelyte N. Serpytis R. (2015). Post-infarction ventricular septal defect: risk factors and early outcomes. Hellenic J. Cardiol. 56, 66–71. 25701974

[B27] Sim N.-L. Kumar P. Hu J. Henikoff S. Schneider G. Ng P. C. (2012). SIFT web server: predicting effects of amino acid substitutions on proteins. Nucleic Acids Res. 40, W452–W457. 10.1093/nar/gks539 22689647 PMC3394338

[B28] Spicer D. E. Hsu H. H. Co-Vu J. Anderson R. H. Fricker F. J. (2014). Ventricular septal defect. Orphanet J. Rare Dis. 9, 144. 10.1186/s13023-014-0144-2 25523232 PMC4316658

[B29] Stenson P. D. Mort M. Ball E. V. Evans K. Hayden M. Heywood S. (2017). The human gene mutation database: towards a comprehensive repository of inherited mutation data for medical research, genetic diagnosis and next-generation sequencing studies. Hum. Genet. 136, 665–677. 10.1007/s00439-017-1779-6 28349240 PMC5429360

[B30] Szklarczyk D. Morris J. H. Cook H. Kuhn M. Wyder S. Simonovic M. (2017). The STRING database in 2017: quality-controlled protein-protein association networks, made broadly accessible. Nucleic Acids Res. 45, D362–D368. 10.1093/nar/gkw937 27924014 PMC5210637

[B31] The 1000 Genomes Project Consortium Auton A. Abecasis G. R. (2015). A global reference for human genetic variation. Nature 526, 68–74. 10.1038/nature15393 26432245 PMC4750478

[B32] The Gene Ontology Consortium (2017). Expansion of the gene ontology knowledgebase and resources. Nucleic Acids Res. 45, D331–D338. 10.1093/nar/gkw1108 27899567 PMC5210579

[B33] Volders P.-J. Anckaert J. Verheggen K. Nuytens J. Martens L. Mestdagh P. (2018). LNCipedia 5: towards a reference set of human long non-coding RNAs. Nucleic Acids Res. 47 (D1), D135–D139. 10.1093/nar/gky1031 PMC6323963 30371849

[B34] Wang K. Li M. Hakonarson H. (2010). ANNOVAR: functional annotation of genetic variants from next-generation sequencing data. Nucleic Acids Res. 38, e164. 10.1093/nar/gkq603 20601685 PMC2938201

[B35] Wei Q. Zhan X. Zhong X. Liu Y. Han Y. Chen W. (2015). A bayesian framework for *de novo* mutation calling in parents-offspring trios. Bioinformatics 31, 1375–1381. 10.1093/bioinformatics/btu839 25535243 PMC4410659

[B36] Wood A. R. Esko T. Yang J. Vedantam S. Pers T, H. Gustafsson S. (2014). Defining the role of common variation in the genomic and biological architecture of adult human height. Nat. Genet. 46, 1173–1186. 10.1038/ng.3097 25282103 PMC4250049

[B37] Zhao L. Chen Q. Li W. Jiang P. Wong L. Li J. (2017). Mapreduce for accurate error correction of next-generation sequencing data. Bioinformatics 33, 3844–3851. 10.1093/bioinformatics/btx089 28205674

[B38] Zhao L. Xie J. Bai L. Chen W. Wang M. Zhang Z. (2018). Mining statistically-solid k-mers for accurate NGS error correction. BMC Genomics 19, 912. 10.1186/s12864-018-5272-y 30598110 PMC6311904

[B39] Zhao Y. Li H. Fang S. Kang Y. Wu W. Hao Y. (2016). NONCODE 2016: an informative and valuable data source of long non-coding RNAs. Nucleic Acids Res. 44, D203–D208. 10.1093/nar/gkv1252 26586799 PMC4702886

